# 2D and 3D In Vitro Culture Systems in Biomedical Research: From Basic Experimental to Translational Utility

**DOI:** 10.3390/ph19071051

**Published:** 2026-07-08

**Authors:** Diego Luis Ribeiro, Ilce Mara de Syllos Cólus, Juliana Mara Serpeloni

**Affiliations:** 1 Department of Microbiology, Institute of Biomedical Sciences, University of São Paulo (ICB/USP), São Paulo 05508-000, São Paulo, Brazil; 2 Department of General Biology, Center of Biological Sciences, State University of Londrina (UEL), Londrina 86057-970, Parana, Brazil; colus@uel.br

## 1. Landscape of Cell Culture Models in Biochemical Research

**Figure 1:**
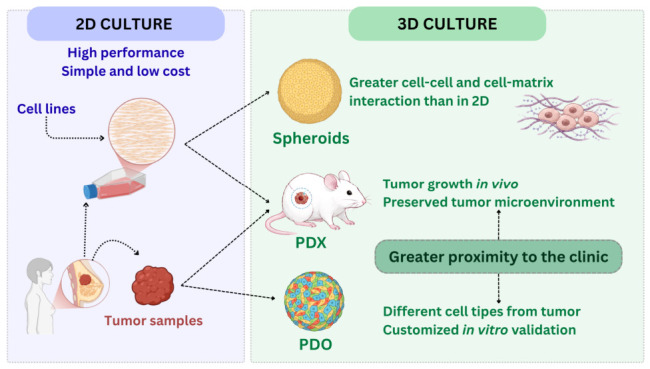
Comparison of preclinical cancer models used in translational research. Conventional two-dimensional (2D) cell culture provides a simple, reproducible, and cost-effective platform for high-throughput screening using immortalized cell lines or patients’ tumor samples. More physiologically relevant models include three-dimensional (3D) spheroids, patient-derived organoids (PDOs), and patient-derived xenografts (PDXs), which progressively recapitulate key features of the patient’s tumor.

Over the past decade, two-dimensional (2D) and three-dimensional (3D) in vitro culture systems have evolved from simple experimental formats into central platforms for mechanistic basic biology, disease modeling, pharmacological testing, and translational research. While in 2D cultures, cells readily grow, forming a monolayer in the flask, numerous technologies are used to generate tumor spheroids or organoids that more closely mimic the characteristics of native tumors and their microenvironment. We cannot say that 2D culture is obsolete, because both models complement each other, depending on the research objective. Rather than framing a duel between “traditional monolayer” and “advanced 3D” in vitro approaches, these systems are better described as complementary tools, each offering advantages and disadvantages depending on the study’s central biological question. This complementarity was highlighted by Motohashi et al. [1], who proposed an integrated workflow for preclinical drug screening in which 2D screening provides high-throughput compound profiling, while 3D models such as patient-derived organoids (PDOs) and patient-derived xenografts (PDXs) offer personalized validation and confirm in vivo efficacy (Figure 1).

In this regard, this research field has moved beyond the question of whether 3D cell culture is superior to 2D culture and now addresses a more important question: which model best balances physiological relevance, experimental tractability, reproducibility, scalability, and translational utility?

This Special Issue, “2D and 3D Culture Systems: Current Trends and Biomedical Applications”, was designed to explore how these two models have been used in cutting-edge research in recent years. The contributions come from research groups in nine countries: five in Asia, three in Europe, and one in North America. The nine peer-reviewed articles, including six original research articles and three comprehensive reviews, cover diverse applications of 2D and 3D culture systems in toxicology, oncology, organoid engineering, disease modeling, regenerative strategies, and disease-relevant platforms.

## 2. An Overview of Contributions of This Special Issue

The contributions gathered here reflect different current applications of in vitro culture technologies in toxicology, oncology, organoid engineering, disease modeling, stem cell-based regenerative strategies, and disease-relevant platforms. Collectively, these studies and reviews reinforce a central message: increasing structural complexity is not, by itself, the ultimate goal. The most important challenge in cell culture today is to develop models that are biologically informative, technically robust, and fit for purpose.

Conventional 2D systems remain indispensable due to their simplicity, accessibility, cost-effectiveness, imaging compatibility, and suitability for high-throughput screening [2]. 2D culture also has well-recognized limitations. Cells grown in 2D monolayers display altered morphology, polarity, proliferation dynamics, and drug responses; moreover, they lack the 3D architecture, diffusion gradients, and multicellular interactions that characterize living tissues [3]. Emerging 3D technologies such as microfluidic systems, bioprinting, and advanced biomaterials are being integrated into 3D cultures, offering more physiologically relevant and reproducible models [4]. These innovations are critical for advancing the field toward more predictive and translationally useful in vitro systems, but they remain underutilized.

Different articles in this Special Issue illustrate that we cannot assume one model is always better than another; this must be demonstrated experimentally, depending on the study’s objective. For example, Wallace et al. [5] analyzed 2D and 3D tissue models and their application for inhalation toxicity assessment; they emphasized that advanced in vitro systems should be validated for a defined context of use and that their translational value depends on fit-for-purpose qualification rather than complexity. This perspective is particularly relevant as regulatory agencies increasingly adopt New Approach Methodologies (NAMs) and seek human-relevant alternatives to traditional in vivo animal testing [6].

In oncology, the contributions reveal the diversity of ways in which 3D systems can clarify tumor behavior while also cautioning against simplistic assumptions about model superiority. Park et al. [7] compared glioblastoma (GBM) culture platforms and found that a tumorsphere liquid-medium platform preserved transcriptional similarity to paired tumor tissue more effectively than more elaborate extracellular-matrix-based conditions. This is a particularly important contribution because it reminds the field that a more complex model is not necessarily more faithful. Other tumor spheroids studied in this Special Issue further show that microenvironmental factors, including extracellular vesicles, can modulate therapeutic response in a cell-type-dependent manner. Such findings underscore the value of 3D tumor models in cancer research by identifying microenvironment-dependent phenotypes that may be flattened or missed entirely in 2D culture.

The growing importance of organoid-based systems is also strongly represented in this Special Issue. The review contributions address stem cell-derived GBM organoids from a bioengineering perspective and next-generation hydrogels as scaffolds for biliary organoid engineering. These discussions align with broader evidence that iPSC- and patient-derived organoids have substantial preclinical potential, yet still face major challenges in scalability, reproducibility, matrix definition, and standardization [8]. Together, these perspectives reinforce the fact that the future of 3D culture science will depend not only on cell biology but also on the biomaterials and scaffold-based or scaffold-free systems used, and, more importantly, on the rigorous standardization of methods.

Another strength of this Special Issue is its emphasis on functionally informative and disease-relevant platforms. Lengacher et al. [9] generated a brain organoid from patient cells with GLUT1 deficiency syndrome to model the link between hypometabolism and epileptiform activity; they provided a human-specific platform for examining disease mechanisms and evaluating therapeutic strategies. This study couples 3D culture (organoid) with a platform containing multielectrode array recordings and quantitative signal analysis, illustrating how advanced functional models can move 3D culture systems beyond basic cell culture models. This combination of cells with a physical architecture that mimics the nervous system is especially promising for neurodegenerative diseases, where network-level behavior and human-specific cellular interactions remain difficult to capture in standard preclinical systems. These integrated systems may provide valuable insights into biomedical research and improve diagnostic and therapeutic approaches for neurodegenerative diseases [10].

This Special Issue shows that some questions are best answered in carefully controlled 2D systems, while others require spheroids or organoids to reveal multicellular or spatially organized responses. In some cases, as shown by Park et al. [7] in a GBM model, 3D cell culture using simple, cost- and time-efficient liquid media to form tumor spheroids may be preferable to complex platforms supplemented with extracellular matrix components, depending on the endpoint. In others, the inclusion of 3D architecture and functionally relevant context is essential for uncovering biologically meaningful differences in treatment response or disease behavior. This is the most important message of this issue: the value of a cell culture model lies not in its complexity per se, but in the alignment of its design with the biological question and its intended use.

## 3. Future Perspectives and Final Considerations

Looking ahead, several directions appear especially important for facilitating future translational applications: (i) continuing the transition toward chemically defined, xeno-free, and tunable matrices; (ii) incorporating multicellular complexity in advanced culture models; (iii) placing greater emphasis on real-time analysis; (iv) adopting explicit reporting standards, cross-platform benchmarking, and context-of-use frameworks in model development; and (v) strengthening the coupling among advanced in vitro systems, computational analysis, patient-derived materials, and clinical datasets. Addressing these points will be essential for improving scalability, reproducibility, and preclinical confidence in organoid and advanced 3D culture platforms [8].

In conclusion, the articles in this Special Issue show that 2D and 3D culture systems are complementary methodological alternatives that enhance the experimental pre-clinical models and are shaping the future of biomedical research. Their continued refinement, including organ-on-chip and tissue-engineered models, is changing how we study disease biology and therapeutic responses and is helping bridge the gap between experimental biology and clinical translation. These systems also allow scientists to generate reliable models of human tissues, organs, and pathologies while reducing reliance on animal testing [11]. We hope this Special Issue stimulates further interdisciplinary collaboration among cell biologists, engineers, pharmacologists, toxicologists, and clinician-scientists, and encourages the development of next-generation in vitro platforms that are more reproducible, interpretable, and useful for biomedical applications.

## References

[1] Motohashi S., Katsuta E., Ban D. (2025). Advances and Challenges in Drug Screening for Cancer Therapy: A Comprehensive Review. Bioengineering.

[2] Kapałczyńska M., Kolenda T., Przybyła W., Zajączkowska M., Teresiak A., Filas V., Ibbs M., Bliźniak R., Łuczewski Ł., Lamperska K. (2018). 2D and 3D Cell Cultures—A Comparison of Different Types of Cancer Cell Cultures. Arch. Med. Sci..

[3] Tibbitt M.W., Anseth K.S. (2009). Hydrogels as Extracellular Matrix Mimics for 3D Cell Culture. Biotechnol. Bioeng..

[4] Sreepadmanabh M., Arun A.B., Bhattacharjee T. (2024). Design Approaches for 3D Cell Culture and 3D Bioprinting Platforms. Biophys. Rev..

[5] Wallace J., McElroy M.C., Klausner M., Corley R., Ayehunie S. (2025). Two-and Three-Dimensional Culture Systems: Respiratory In Vitro Tissue Models for Chemical Screening and Risk-Based Decision Making. Pharmaceuticals.

[6] U.S. Food and Drug Administration General Considerations for the Use of New Approach Methodologies in Drug Development. https://www.fda.gov/regulatory-information/search-fda-guidance-documents/general-considerations-use-new-approach-methodologies-drug-development.

[7] Park J., Koh I., Cha J., Oh Y., Shim J.-K., Kim H., Moon J.H., Kim E.H., Chang J.H., Kim P. (2024). Comparison of Glioblastoma Cell Culture Platforms Based on Transcriptional Similarity with Paired Tissue. Pharmaceuticals.

[8] Heinzelmann E., Piraino F., Costa M., Roch A., Norkin M., Garnier V., Homicsko K., Brandenberg N. (2024). iPSC-Derived and Patient-Derived Organoids: Applications and Challenges in Scalability and Reproducibility as Pre-Clinical Models. Curr. Res. Toxicol..

[9] Lengacher L., Lengacher S., Magistretti P.J., Finsterwald C. (2026). GLUT1-DS Brain Organoids Exhibit Increased Sensitivity to Metabolic and Pharmacological Induction of Epileptiform Activity. Pharmaceuticals.

[10] Amartumur S., Nguyen H., Huynh T., Kim T.S., Woo R.-S., Oh E., Kim K.K., Lee L.P., Heo C. (2024). Neuropathogenesis-on-Chips for Neurodegenerative Diseases. Nat. Commun..

[11] Bédard P., Gauvin S., Ferland K., Caneparo C., Pellerin È., Chabaud S., Bolduc S. (2020). Innovative Human Three-Dimensional Tissue-Engineered Models as an Alternative to Animal Testing. Bioengineering.

